# Bioactive Chitosan-Alginate Films with Oregano or Turmeric: Effects on Blueberry Shelf Life and In Vitro Genotoxicity Profile

**DOI:** 10.3390/molecules31162751

**Published:** 2026-08-07

**Authors:** Aleksandra Such, Ewelina Węsierska, Magdalena Surma, Anna Winiarz, Aneta Koronowicz

**Affiliations:** 1 Department of Human Nutrition and Dietetics, Faculty of Food Technology, University of Agriculture in Krakow, Balicka 122, 30-149 Krakow, Poland; winiarz.am@gmail.com; 2 Department of Chemistry, Faculty of Food Technology, University of Agriculture in Krakow, Balicka 122, 30-149 Krakow, Poland; 3 Department of Infectious Diseases and Public Health, Faculty of Veterinary Medicine, Rędzina 1 C, 30-248 Krakow, Poland; ewelina.wesierska@urk.edu.pl; 4 Department of Plant Products Technology and Nutrition Hygiene, Malopolska Centre of Food Monitoring, Faculty of Food Technology, University of Agriculture in Krakow, Balicka 122, 30-149 Krakow, Poland; magdalena.surma@urk.edu.pl

**Keywords:** chitosan-alginate films, bioactive packaging films, oregano extract, turmeric extract, blueberry shelf life, antioxidant activity, genotoxicity, food safety

## Abstract

Synthetic plastics remain widely used in food packaging despite environmental concerns. This laboratory-scale study evaluated biodegradable chitosan-alginate films enriched with oregano or turmeric two-phase equilibrium condensates as active packaging films for highbush blueberries (*Vaccinium corymbosum* L.). Film hydration properties were assessed, and blueberries were stored at 4 °C or 22 °C for 7 or 14 days. Dry matter, proximate composition, total phenolic content (TPC), antioxidant activity, vitamin C, and total viable bacterial count (TVC) were measured. Simulated gastrointestinal film digestates were evaluated in Nthy-ori 3-1 thyroid epithelial and CCD 841 CoN colonic epithelial cells. Oregano-enriched films (OS3) showed greater water uptake and swelling than control and turmeric-enriched films. Blueberry responses depended on formulation and storage conditions. After 14 days at 4 °C, OS2- and OS3-packaged blueberries showed higher vitamin C contents than plastic controls, corresponding to approximately 20% lower vitamin C loss. TVC was primarily influenced by temperature, with no significant main effect of packaging type. Digestates did not significantly alter evaluated DNA-damage markers under short-term exposure conditions. Concentration-dependent cell-cycle changes occurred in Nthy-ori 3-1 cells after OS2 and OS3 exposure, whereas no significant changes were observed in CCD-841CoN cells.

## 1. Introduction

Synthetic plastics dominate food packaging, yet microplastic pollution in ecosystems and food chains necessitates biodegradable alternatives [1,2]. Chitosan-alginate polyelectrolyte complexes, derived from renewable polysaccharides, provide edible films with enhanced barrier properties (moisture, gases) and function as carriers for controlled release of active phytochemicals, antimicrobials, and antioxidants, via direct contact and headspace diffusion at the food surface.

Chitosan, obtained by deacetylation of chitin, is one of the most abundant biopolymers and provides film-forming ability, biocompatibility, and biodegradability, primarily serving as a polycationic carrier for antimicrobial and antioxidant bioactives when complexed with alginate [3,4,5].

Sodium alginate, a naturally occurring anionic polysaccharide isolated from brown algae, exhibits excellent film-forming capability, gelation in the presence of multivalent cations, and high oxygen-barrier properties, and it is widely used as a structural component of edible coatings [5,6]. In chitosan-alginate systems, electrostatic interactions between cationic amino groups in chitosan and anionic carboxylate groups in alginate yield polyelectrolyte complexes with improved mechanical strength and stability relative to the individual polymers [5]. These complexes form transparent, flexible films that are generally regarded as safe for food contact and can act as carriers and controlled-release matrices for a wide range of bioactive compounds [1,5].

Incorporation of natural plant-derived compounds, particularly polyphenols and essential oils, into such biopolymer matrices enables the design of active packaging systems that do more than passively separate food from the environment. Polyphenols are capable of scavenging free radicals, chelating pro-oxidant metal ions, and modulating enzymatic pathways involved in quality degradation, while essential oils provide broad-spectrum antimicrobial activity [1,7]. Oregano (*Origanum vulgare* L.) essential oil is rich in phenolic monoterpenes such as carvacrol and thymol, which can interact with the lipid components of microbial membranes, increase membrane fluidity and permeability, collapse the proton motive force and ultimately lead to cell death [7,8]. These compounds have demonstrated activity against both Gram-positive and Gram-negative bacteria and various spoilage fungi, and their efficacy can be further enhanced when immobilized or entrapped in polymeric films that provide gradual release at the food surface [5,7,8].

Turmeric (*Curcuma longa* L.), in turn, provides a curcuminoid-rich extract in which curcumin is the major component. Curcumin is a polyphenolic diarylheptanoid characterized by an extensive conjugated π-system and phenolic hydroxyl groups that confer potent antioxidant activity through hydrogen-atom transfer, single-electron transfer and metal chelation mechanisms [9,10]. Its high hydrophobicity and tendency to self-associate result in low aqueous solubility and limited stability in biological and food matrices [9,10]. However, immobilization within hydrophilic–hydrophobic polymer networks and various nanostructured carriers has been shown to improve its apparent solubility, chemical stability, and controlled release, extending its functional lifetime as an antioxidant and, in some systems, as an antimicrobial agent [9,10]. Embedding turmeric extracts in chitosan-alginate matrices may provide an approach for developing edible films with enhanced radical-scavenging capacity and potential applications in limiting oxidative deterioration in high-value fruits [5].

Highbush blueberries are an excellent model for testing such active packaging systems due to their recognized health benefits, high sensitivity to postharvest damage, and intensive global trade. Blueberries are classified as a superfruit owing to their high content of anthocyanins, flavonols, phenolic acids and ascorbic acid, which together contribute substantial antioxidant capacity and are associated with a wide range of biological effects relevant to human health [11,12]. Blueberries contain a complex mixture of anthocyanins, primarily malvidin, delphinidin, cyanidin, petunidin and peonidin glycosides, alongside chlorogenic acid and quercetin derivatives, and these phytochemicals exhibit strong free-radical-scavenging, anti-inflammatory and cardioprotective activities. From a technological perspective, these compounds are also key quality biomarkers: their retention during storage reflects the extent of oxidative and enzymatic degradation, while changes in anthocyanin composition are closely linked to color stability and consumer acceptance [12,13].

Despite the recognized importance of their bioactive profile, fresh blueberries are highly susceptible to mechanical injury, water loss, softening, and fungal pathogens such as *Botrytis cinerea* and *Glomerella cingulata*, as well as bacterial growth during handling, storage and distribution [12,14]. Recent work has demonstrated that chitosan-based interventions can effectively improve postharvest quality. Figiel-Kroczyska et al. [14] reported that preharvest spraying of highbush blueberry with chitosan solutions of varying molecular weight significantly improved physical and biochemical attributes after harvest. High-molecular-weight chitosan (125–500 kDa) increased mean berry weight and firmness, enhanced ascorbic acid and total polyphenol content, elevated antioxidant activity (ABTS, FRAP, DPPH) and reduced fungal contamination and mycotoxin occurrence, suggesting that chitosan can act both as a plant elicitor and as a direct antimicrobial barrier on the fruit surface [14]. Other studies on edible coatings for berries and soft fruits have similarly shown that chitosan-based and polysaccharide-based coatings can reduce respiration rate and water loss, delay softening and browning, and suppress surface microflora, thereby extending shelf life under refrigerated storage [1,15].

Building on these observations, our previous study demonstrated the development and characterization of chitosan-alginate films enriched with oregano essential oil and turmeric extract as edible coatings for tofu [5]. We showed that incorporation of these botanical agents significantly enhanced thermal and mechanical properties, increasing tensile strength and elongation at break in oregano-containing films while improving thermal stability by ~10 °C. These coatings exhibited pronounced antimicrobial activity on tofu and proved non-cytotoxic in vitro: no IC_50_ was reached in HepG2 and BJ cell models at maximum extract concentrations, with proliferative effects observed under certain conditions. These results established the safety of oregano- and turmeric-enriched alginate-chitosan films for food-contact applications and confirmed their suitability as functional active packaging materials [5].

While our previous work confirmed the structural integrity and baseline non-cytotoxicity of these specific films, translating such biopolymer matrices into practical food packaging requires evaluating their functional behavior under realistic environmental stress. First, it is important to clearly distinguish between the well-documented antimicrobial and antioxidant activities of oregano and turmeric when evaluated as independent agents, and the still underexplored behavior of these botanicals when immobilized within chitosan-alginate matrices. Currently, a major gap exists in understanding how the incorporation of chemically distinct biphasic condensates alters the hydration and swelling dynamics of the chitosan-alginate network, and how these physical structural changes, combined with the release of volatile bioactives, dictate the preservation of high-moisture, perishable fruits. Moreover, investigating active packaging performance across different thermal conditions, such as strict cold chain versus ambient storage, provides a more comprehensive understanding of their practical efficacy, given that temperature strongly influences both bioactive compound release and fruit preservation. Crucially, advancing the safety profile of novel food-contact materials demands moving beyond basic cell viability. Thus, assessing the potential genotoxicity of the complex simulated gastrointestinal digestates from the entire film provides a more realistic safety model compared to testing the pure botanical extracts alone.

There is extensive evidence that oregano essential oil and turmeric extracts exhibit strong antimicrobial and antioxidant activities independently, and previous research has demonstrated that turmeric extract can modify the moisture content and hydration behavior of polymer films [16]. Building on this foundation, we hypothesized that when immobilized within a cross-linked chitosan-alginate matrix, these bioactives would play distinct functional roles. Specifically, we hypothesized that the dynamic release of bioactive volatiles from oregano-enriched films would primarily enhance the antimicrobial and antioxidant protection of packaged blueberries, whereas the highly hydrophobic turmeric condensates would exert a stronger influence on the physical stability of the matrix itself, thereby better preserving the fruit’s basic composition. Furthermore, we hypothesized that both film formulations would maintain a favorable, non-genotoxic profile at relevant food-contact exposures.

The present study evaluated solution-cast chitosan-alginate films enriched with oregano or turmeric condensates as laboratory-scale active packaging for blueberries. The primary aim was to assess fruit quality changes during storage, supported by material characterization and in vitro safety tests. To achieve this, the specific objectives were divided into three key areas. First, we characterized film hydration properties (moisture content, water uptake, swelling degree) to understand how polyphenolic partitioning influences swelling dynamics and bioactive release. Second, we examined the effects of packaging formulation, storage temperature (4 °C cold chain vs. 22 °C ambient abuse for 7 days), and storage time (7 and 14 days) on blueberry quality. This comprehensive assessment included dry matter, proximate composition, total phenolic and ascorbic acid content, antioxidant activity (ABTS, DPPH), and total viable bacterial count. Finally, building on our previous cytotoxicity studies, we screened the genotoxicity of simulated gastrointestinal digestates of the films. This was performed in Nthy-ori 3-1 thyroid and CCD 841 CoN colonic epithelial cells using exposure levels (0.01–100 µg/mL) scaled to migration estimates for an approximate 32 m^2^ human intestinal surface area.

## 2. Results and Discussion

### 2.1. Bioactive Film Characterization

#### 2.1.1. Appearance, Homogeneity, and Moisture Content

**Figure 1:**
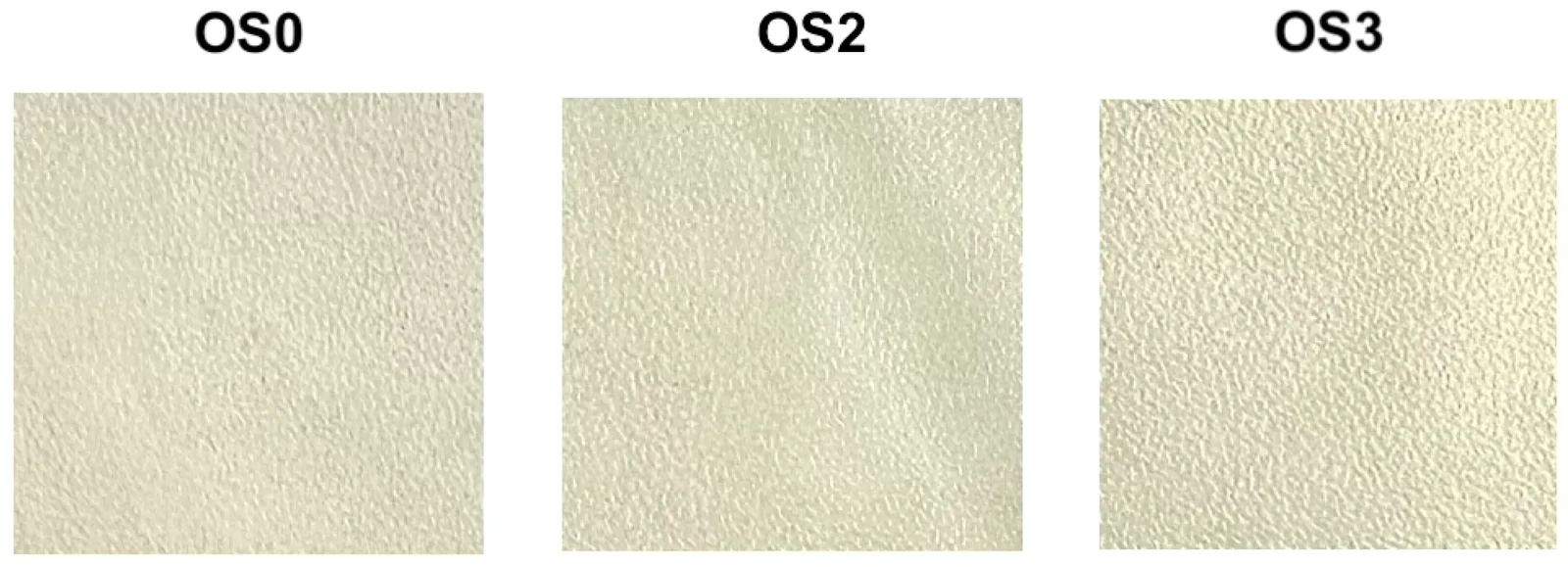
Macroscopic appearance of solution-cast chitosan-alginate films: OS0 (control), OS2 (turmeric-enriched), and OS3 (oregano-enriched).

All chitosan-alginate films formed continuous, flexible sheets without visible cracks, pinholes, or phase separation, which is a fundamental prerequisite for their application as food packaging materials (Figure 1). As demonstrated by the macroscopic surface morphology, all formulations exhibited a consistent, pale yellowish-cream optical profile characterized by uniform micro-texturing, which is a typical phenomenon resulting from the solution-casting and solvent evaporation processes.

The incorporation of the bioactive biphasic condensates did not visibly alter the macroscopic appearance of the films. Compared with the control film (OS0), the turmeric-enriched (OS2) and oregano-enriched (OS3) formulations showed no visible cracks, pinholes, phase separation, or sedimented aggregates. These observations indicate acceptable macroscopic homogeneity under the applied preparation conditions. However, microscopic characterization would be required to confirm the distribution of the condensates within the polymer matrix.

**Figure 2:**
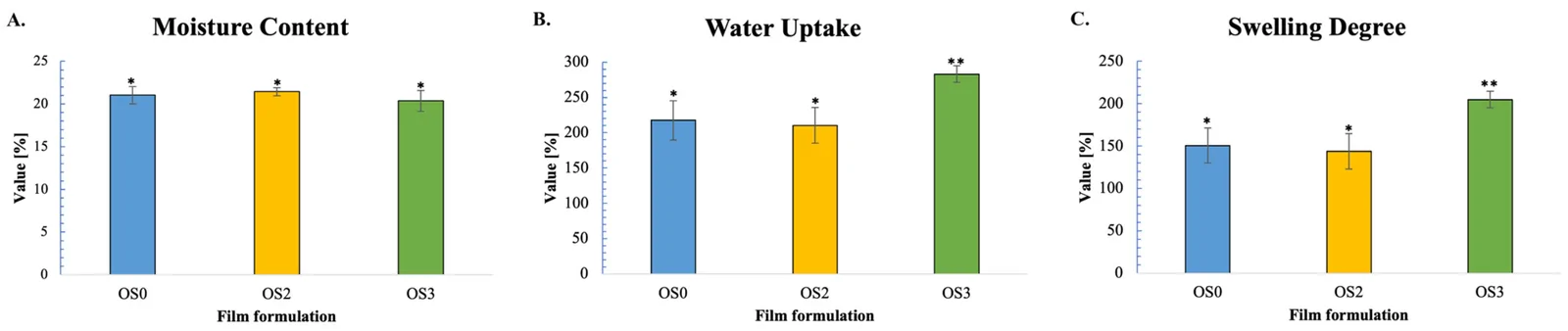
Hydration properties of chitosan-alginate bioactive films. Moisture content (**A**), 24 h water uptake capacity (**B**), and swelling degree (**C**) of control (OS0), turmeric-enriched (OS2), and oregano-enriched (OS3) chitosan-alginate films. Data represent as means ± SD (n = 5). Asterisks indicate statistically significant differences among formulations: *p* < 0.05 = *, *p* < 0.01 = ** (one-way ANOVA with Tukey post hoc test).

Moisture content analysis demonstrated minimal compositional variation across all three film formulations (Figure 2A). OS0 control films exhibited 21.0 ± 1.0% moisture content, while OS2 (turmeric-enriched) and OS3 (oregano-enriched) films displayed 21.4 ± 0.5% and 20.4 ± 1.2%, respectively. One-way ANOVA revealed no statistically significant differences (*p* > 0.05), indicating that botanical extract incorporation did not substantially alter the baseline hydration capacity of the dried chitosan-alginate matrix.

The moisture contents of all formulations were within the range reported for comparable polysaccharide-based films [17]. The absence of significant differences among OS0, OS2, and OS3 indicates that incorporation of the condensates did not measurably affect the equilibrium moisture content of the dried films under the applied conditions.

Although essential oils and phenolic-rich extracts may alter the moisture content of biopolymer films, no significant differences in equilibrium moisture content were observed among the formulations in the present study. One possible explanation is that the chitosan-alginate matrix remained the dominant determinant of water retention after drying, thereby limiting the measurable contribution of the incorporated condensates. However, this interpretation is hypothetical because the retention, distribution, and molecular interactions of the condensate components in the dried films were not directly determined. Future compositional and structural analyses would be required to verify this explanation.

#### 2.1.2. Water Uptake and Swelling Degree

In striking contrast to moisture content, water uptake capacity demonstrated substantial and highly significant compositional dependence (Figure 2B). OS0 control films absorbed 217.6 ± 27.7% water, OS2 (turmeric biphasic condensate) films exhibited 210.3 ± 25.2%, while OS3 (oregano biphasic condensate) films achieved markedly elevated water uptake of 283.0 ± 11.7% after 24 h aqueous immersion (Figure 2B). ANOVA analysis confirmed statistically significant differences among formulations (F = 15.6, *p* = 4.60 × 10^−4^, *** *p* < 0.001). Post hoc Tukey testing demonstrated that OS3 films exhibited significantly higher water uptake compared to both OS0 (Δ = 65.4%, *** *p* < 0.001) and OS2 formulations (Δ = 72.7%, *** *p* < 0.001), whereas OS0 and OS2 showed no significant difference (*p* > 0.05).

The 30.1% higher water uptake of the oregano-enriched films compared with the control is consistent with previous reports showing increased liquid absorption in oregano-containing chitosan-alginate films [1]. A plausible explanation is that incorporation of the oregano condensate modified the organization of the polymer network and increased its accessibility to water. Such an effect could involve changes in intermolecular interactions between the matrix components and phenolic constituents of the condensate. However, because no spectroscopic or microstructural analysis was performed after condensate incorporation, this mechanism remains hypothetical and should be verified in future studies.

In contrast, the turmeric-enriched films did not differ significantly from the control in water uptake (210.3 ± 25.2% for OS2 vs. 217.6 ± 27.7% for OS0), representing only a 3.3% reduction. One possible explanation is that the turmeric condensate had a limited effect on the water-accessible domains of the chitosan-alginate network under the tested conditions. Considering the generally hydrophobic character of curcuminoids, differences in the composition of the oregano and turmeric condensates may have contributed to their distinct hydration-related behavior. Nevertheless, the composition, retention, and distribution of the individual bioactive constituents in the dried films were not determined; therefore, this explanation should be regarded as a hypothesis requiring direct verification.

Swelling degree analysis mirrored water uptake trends, demonstrating pronounced compositional effects (Figure 2C). OS0 control films exhibited swelling of 150.7 ± 20.6% and OS2 films displayed similar expansion of 143.9 ± 20.7%, whereas OS3 films showed substantially greater dimensional increase of 204.9 ± 9.7%, equivalent to a 35.9% rise relative to controls (Figure 2C). ANOVA confirmed significant differences across formulations (F = 17.72, *p* = 2.62 × 10^−4^, *** *p* < 0.001), with post hoc analysis revealing that OS3 swelling significantly exceeded both OS0 (*** *p* < 0.001) and OS2 (*** *p* < 0.001).

The higher swelling of the oregano-enriched films was consistent with their higher water uptake. Similar hydration-related effects have been reported for phenolic-modified biopolymer films and have been attributed to changes in polymer-network interactions [7,8,17,18,19,20]. Thus, incorporation of the oregano condensate may have altered the hydration behavior of the chitosan-alginate matrix. However, because molecular interactions and structural changes were not directly assessed, this explanation remains a literature-based hypothesis. The lower variability observed for OS3 should also be interpreted cautiously.

The distinct hydration and swelling profiles of the films may be relevant to their performance as active packaging materials. The higher swelling observed for OS3 indicates a greater response of this formulation to aqueous exposure; however, its implications for the release of oregano-derived compounds were not determined in the present study. Because the films were used as macro-perforated pouches, their overall package performance was influenced not only by the intrinsic properties of the film matrix but also by the perforation design. Accordingly, no direct conclusions can be drawn regarding the relationship between swelling, barrier performance, or active-compound release. Further studies combining migration, release, and structural analyses are needed to clarify these relationships.

### 2.2. Blueberry Preservation During Storage

#### 2.2.1. Dry Matter Content and Proximate Composition

Blueberry dry matter content was affected by packaging type, storage temperature, and storage duration (Table 1; three-way ANOVA, *p* < 0.05). The initial dry matter content was 12.13 ± 1.22%. After 7 days of storage at 4 °C, no significant increase was observed. In contrast, the highest dry matter content was recorded in blueberries packed in OS0 after 7 days at 22 °C (16.69 ± 0.74%), which was significantly higher than the initial value (Tukey’s test, *p* < 0.05). This increase is consistent with greater water loss under higher-temperature storage conditions. Under the same conditions, blueberries packed in OS2 showed a lower dry matter content (13.93 ± 1.32%), suggesting that the turmeric-enriched formulation may have attenuated storage-related changes in dry matter. Although the basis of this response was not established in the present study, it may be related to differences in the fruit-package microenvironment [21]. Higher dry matter % serves as a standard proxy for weight loss in postharvest studies.

Protein, dietary fiber, and ash contents did not show consistent treatment-dependent changes during storage (Table 1). The observed variability did not support a clear effect of packaging formulation on these parameters.

Lipid content ranged from 0.59 to 0.94 g/100 g dry weight across the experimental groups (Table 1). After 7 days of storage, OS2-packed blueberries showed the highest lipid values under both temperature conditions. At 4 °C, the OS2 value did not differ significantly from that of the commercial packaging (Cp), whereas both treatments showed higher values than OS0. At 22 °C, OS2 showed a significantly higher lipid content than both Cp and OS0 (*p* < 0.05). After 14 days at 4 °C, no significant difference was observed between OS2 and OS0. Overall, the OS2 formulation was associated with higher lipid values during short-term storage, particularly at 22 °C. As lipid oxidation was not measured, these differences cannot be attributed to a specific antioxidant or protective mechanism.

Overall, the turmeric-enriched chitosan-alginate film (OS2) was associated with more favorable values for selected compositional parameters under specific storage conditions, particularly during short-term storage. The most evident differences were observed for lipid content, whereas protein, dietary fiber, and ash did not show consistent treatment-dependent changes. These findings indicate a potential contribution of the OS2 formulation to the preservation of selected blueberry quality attributes; however, this effect was not uniform across all parameters and storage conditions.

#### 2.2.2. Total Phenolic Content, Ascorbic Acid, and Radical Scavenging Activity

Total phenolic content (TPC) increased during storage in all treatments, including the plastic control (Cp) (Table 2); therefore, this increase should not be interpreted as a direct improvement in fruit quality. It may reflect changes in dry matter concentration and/or phenolic extractability during storage. Under refrigerated conditions, TPC values remained closer to the initial level across treatments, whereas higher values were observed after 7 days at 22 °C. The highest TPC value was recorded for OS3 under ambient storage conditions. This observation is consistent with previous reports suggesting that chitosan-based treatments may influence phenolic content in highbush blueberries [14,21,22]. Although curcumin/chitosan systems have been associated with protection against oxidative deterioration in other experimental models [23,24], no consistent packaging-related pattern in TPC was observed under refrigerated storage in the present study.

The two antioxidant assays showed different patterns. The highest ABTS activity was recorded for OS3 after 7 days at 22 °C (237.40 ± 12.81 µmol TE/g dry weight), whereas the highest DPPH activity was observed for OS2 under the same conditions (106.28 ± 1.57 μmol TE/g dry weight) (Table 2). Under refrigerated storage, no consistent differences among packaging treatments were observed. Previous studies have indicated that bioactive packaging may influence antioxidant-related parameters during postharvest storage [22].

Vitamin C content declined across all treatments from the initial value of 25.71 mg/100 g fresh weight to 11.44–14.31 mg/100 g fresh weight after 14 days of storage. After 14 days of refrigerated storage at 4 °C, blueberries packaged in the chitosan-alginate active pouches showed higher vitamin C contents than those stored in the plastic control (Cp). Vitamin C contents were 14.31 mg/100 g fresh weight for OS2 and 13.43 mg/100 g fresh weight for OS3, compared with 11.44 mg/100 g fresh weight for Cp. Under this specific storage condition, the higher vitamin C contents in the OS2- and OS3-packaged blueberries corresponded to an approximately 20% lower vitamin C loss relative to the plastic control. This pattern was not consistent across all tested storage conditions. Higher storage temperature was associated with lower vitamin C contents across all packaging treatments.

Although the mechanisms underlying the higher vitamin C retention in the active-pouch treatments were not investigated, this pattern may be related to the antioxidant properties of the chitosan-alginate matrix and incorporated plant-derived constituents, as suggested for comparable systems [14,21,22].

Overall, antioxidant-related parameters varied with storage temperature, duration, and assay type. OS3 showed the highest ABTS activity after 7 days at 22 °C, whereas OS2 was associated with higher vitamin C retention after 14 days under refrigerated storage. These findings suggest that the formulations may influence selected quality-related parameters under the tested laboratory conditions, but they do not demonstrate a uniform improvement in blueberry quality across all treatments.

#### 2.2.3. Total Viable Bacterial Count

Total viable count (TVC) of highbush blueberries increased with storage time (*p* < 0.01), from 2.76 log CFU/g at day 0 to 5.11 log CFU/g by day 14, irrespective of packaging (Table 3). Refrigerated storage (4 °C) maintained lower TVC (3.51 log CFU/g) than 22 °C (4.21 log CFU/g), indicating that temperature was the primary factor associated with microbial growth (Table 3). Packaging type showed no significant main effect on TVC (*p* > 0.05) (Table 3). Significant packaging type × storage time, temperature × storage time, and packaging type × temperature × storage time interactions (all *p* < 0.01) indicated that TVC responses varied across combinations of the tested conditions (Table 3). However, because the main effect of packaging type and pairwise differences among packaging treatments were not significant, these interactions do not demonstrate an antimicrobial effect of the active films.

**Figure 3:**
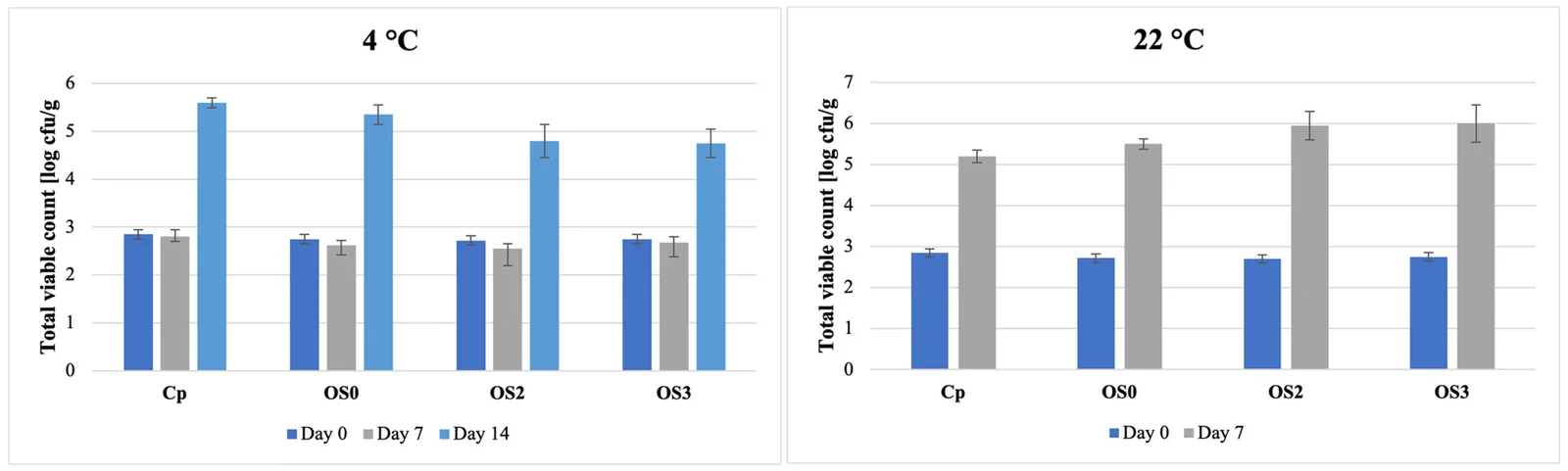
Effect of packaging type, storage temperature, and duration on total viable bacterial count (TVC) (log CFU g^−1^) in highbush blueberries (*Vaccinium corymbosum* L.). Refrigerated storage (4 °C): 7 days and 14 days. Ambient storage (22 °C): 7 days. Data represent means ± SD (n = 3). Different letters indicate significant differences (*p* < 0.05) among packaging types within each time point (one-way ANOVA with Tukey HSD post hoc test). Cp: conventional macro-perforated plastic packaging; OS0: plain chitosan-alginate film; OS2: turmeric-enriched film; OS3: oregano-enriched film.

At 4 °C, OS2 and OS3 showed a modest tendency toward lower TVC than Cp at the evaluated time points (Figure 3); however, these differences were not statistically significant (*p* > 0.05), consistent with the lack of a packaging main effect. At 22 °C, Cp marginally outperformed the bioactive films at day 7 (Figure 3), but again the between-packaging differences were non-significant (*p* > 0.05) and should not be over-interpreted. As our proof-of-concept design utilized macro-perforated pouches for fruit ventilation, microbial dynamics were primarily governed by headspace microclimate and intrinsic fruit physiology rather than physical vapor barrier mechanisms. Overall, storage temperature dominated microbial dynamics (*p* < 0.01), while the significant interaction terms (packaging×time, temperature×time, and packaging×temperature×time; all *p* < 0.01) indicate context-dependent behavior without demonstrating consistent superiority of any packaging across conditions.

The time-dependent TVC increase aligns with microbial succession patterns reported by Wang et al. [25], where 25 °C storage enriched spoilage fungi (*Aspergillus*, *Talaromyces*) while 4 °C maintained diverse bacterial consortia with higher Chao1 indices. Temperature effects dominated packaging mains, consistent with Li et al. [26], who found chitosan films alone insufficient for >2 log CFU/g reductions without nano-enhancers. Significant interactions observed here indicate the conditional efficacy of these bioactive films.

### 2.3. Cell Cycle Analysis

**Figure 4:**
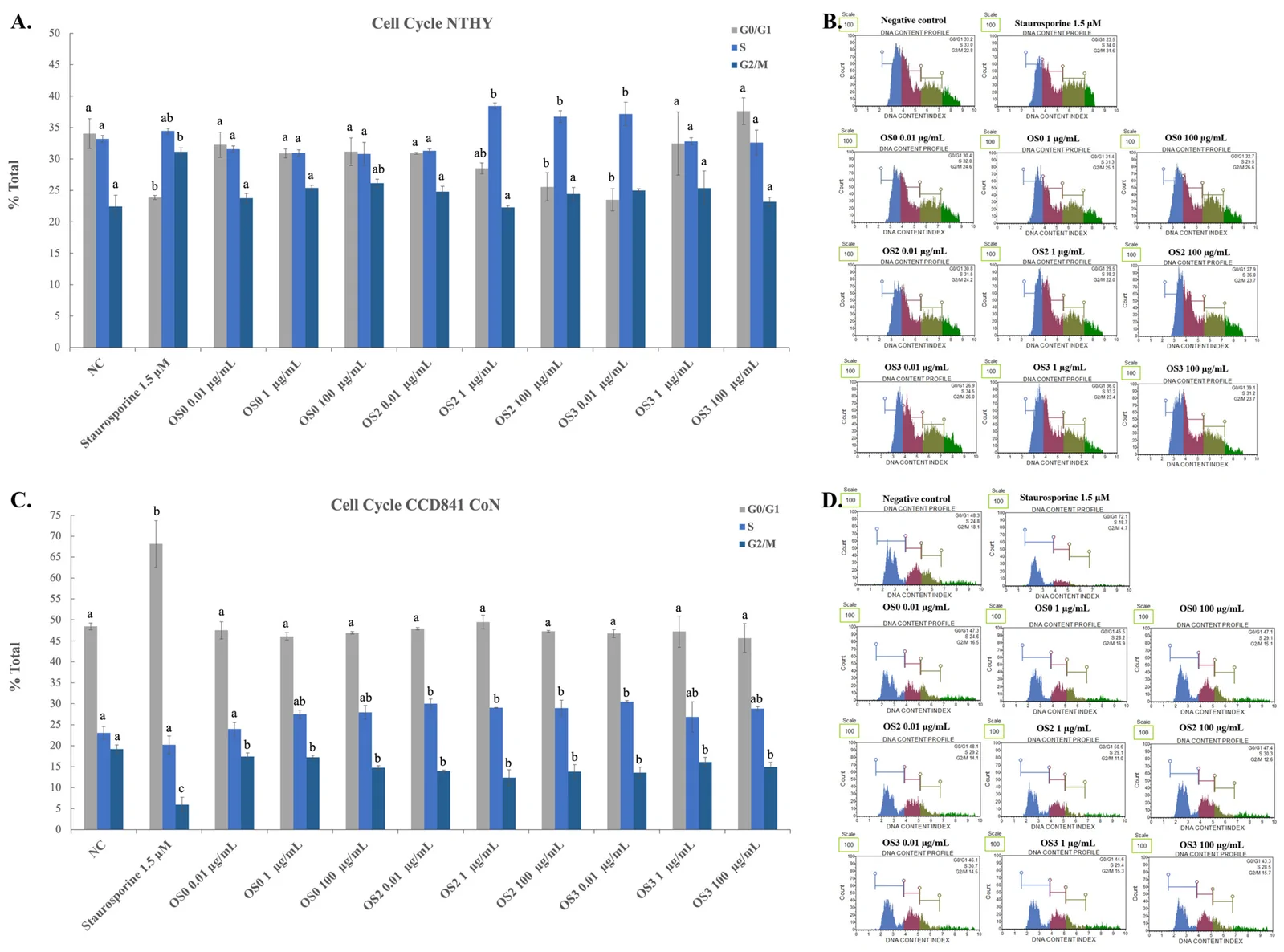
Cell-cycle distribution profiles in Nthy-ori 3-1 (NTHY) thyroid epithelial cells (**A**,**B**) and CCD 841 CoN normal human colonic epithelial cells (**C**,**D**) following exposure to chitosan-alginate film digestates. (**A**,**C**) Representative flow cytometry histograms depicting G0/G1 (blue peaks), S-phase (red peaks), and G2/M (light green peaks) distributions for untreated controls (NC), staurosporine positive control (1.5 µM), and digestates from OS0, OS2, and OS3 films at concentrations of 0.01, 1, and 100 µg/mL. (**B**,**D**) Quantitative phase distributions determined by Muse Cell Analyzer (mean ± SD, n = 3). Values followed by the same letters are not significantly different at *p* < 0.05 (one-way ANOVA with Tukey post hoc test). NC—untreated control; OS0—chitosan:alginate (1:0.25 *w*/*w*) base film digestate; OS2—chitosan:alginate:turmeric (1:0.25:2 *w*/*w*) film digestate; OS3—chitosan:alginate:oregano (1:0.25:1 *w*/*w*) film digestate.

Untreated control cells (NC) showed different cell-cycle distributions in the two cell lines (Figure 4). Nthy-ori 3-1 thyroid epithelial cells showed 34.03 ± 2.36% of cells in the G0/G1 phase, 33.20 ± 0.53% in the S phase, and 22.43 ± 1.78% in the G2/M phase (Figure 4A,B). In CCD 841 CoN colonic epithelial cells, 48.43 ± 0.81% of cells were in G0/G1 phase, while the proportions of cells in S and G2/M phases were 23.03 ± 1.55% and 19.20 ± 0.98%, respectively (Figure 4C,D). The proportion of CCD 841 CoN cells in G0/G1 phase was significantly higher than that observed in Nthy-ori 3-1 cells (*p* < 0.001) (Figure 4).

Staurosporine, used as a positive control, induced significant changes in cell-cycle distribution in both cell lines, confirming assay responsiveness (Figure 4). In Nthy-ori 3-1 cells, staurosporine decreased the proportion of cells in G0/G1 phase to 23.87 ± 0.32% (*p* < 0.05 vs. NC) and increased the proportion of cells in G2/M phase to 31.13 ± 0.64% (Figure 4A,B). The S-phase population was 34.47 ± 0.42% (Figure 4A,B). In CCD 841 CoN cells, staurosporine increased the proportion of cells in G0/G1 phase to 68.15 ± 5.59% (*p* < 0.001 vs. NC), while the proportions in S and G2/M phases decreased to 20.20 ± 2.12% and 5.95 ± 1.77%, respectively (Figure 4C,D).

Exposure to OS0 digestates (0.01–100 µg/mL) did not significantly affect cell-cycle distribution in either cell line (Tukey post hoc test, *p* > 0.05) (Figure 4). Therefore, under the experimental conditions applied, the chitosan-alginate base-matrix digestate did not induce detectable alterations in cell-cycle distribution. This observation is consistent with the absence of cytotoxic effects reported previously for related chitosan-alginate film formulations [5,27].

One-way ANOVA showed significant differences in cell-cycle distribution in Nthy-ori 3-1 cells exposed to OS2 and OS3 digestates (*p* < 0.01), whereas no significant overall effect was observed in CCD 841 CoN cells (*p* = 0.338) (Figure 4). In Nthy-ori 3-1 cells, OS2 exposure was associated with concentration-dependent changes in the proportions of cells in the G0/G1 and S phases (Figure 4A,B). At 0.01 µg/mL, no significant differences from the negative control were observed (G0/G1: 30.90 ± 0.14%; S: 31.30 ± 0.28%; *p* > 0.05) (Figure 4A,B). At 1 µg/mL, the proportion of cells in the G0/G1 phase decreased to 28.50 ± 0.87% (*p* < 0.05), whereas the proportion in the S phase increased to 38.43 ± 0.49% (*p* < 0.05) (Figure 4A,B). At 100 µg/mL, the G0/G1 population further decreased to 25.57 ± 2.25% (*p* < 0.01), while the S-phase population remained elevated at 36.73 ± 0.95% (*p* < 0.05) (Figure 4A,B). Comparable changes in cell-cycle-related parameters have been reported for curcumin-containing chitosan-based systems in other in vitro models [28,29].

OS3 induced concentration-dependent changes in the cell-cycle distribution of Nthy-ori 3-1 cells (Figure 4A,B). At 0.01 µg/mL, the proportion of cells in G0/G1 phase decreased to 23.50 ± 1.75% (*p* < 0.05), whereas the S-phase population increased to 37.17 ± 1.87% (*p* < 0.01) (Figure 4A,B). No significant differences from the negative control were observed at 1 µg/mL (Figure 4A,B). At 100 µg/mL, the proportion of cells in G0/G1 phase increased to 37.60 ± 2.12% (*p* < 0.05), while the S-phase population was 32.60 ± 1.98% (Figure 4A,B). This non-linear pattern indicates that the response to OS3 depended on the tested concentration. The observed response may be related to the effects of essential-oil constituents on cell proliferation and cell-cycle regulation that have been reported in other in vitro systems, including potential involvement of pathways associated with oxidative stress and cell-cycle regulatory proteins [5,30]. However, these mechanisms were not examined in the present study and should therefore be considered only as possible explanations requiring further investigation.

In CCD 841 CoN cells, neither OS2 nor OS3 induced statistically significant changes in cell-cycle distribution at the tested concentrations (maximum change in the S-phase population: 7.47 percentage points; *p* > 0.05) (Figure 4C,D). Thus, the response to the film digestates differed between the thyroid- and colon-derived cell lines. Variable responses to chitosan- and curcumin-containing systems have been reported across cell types and experimental conditions [5,27,28,29]. Nevertheless, the relevance of the cell-line-dependent differences observed here should be interpreted cautiously because no significant changes were detected in CCD 841 CoN cells, and cellular uptake as well as the migration and release of film constituents were not evaluated.

### 2.4. DNA Damage Assessment

**Figure 5:**
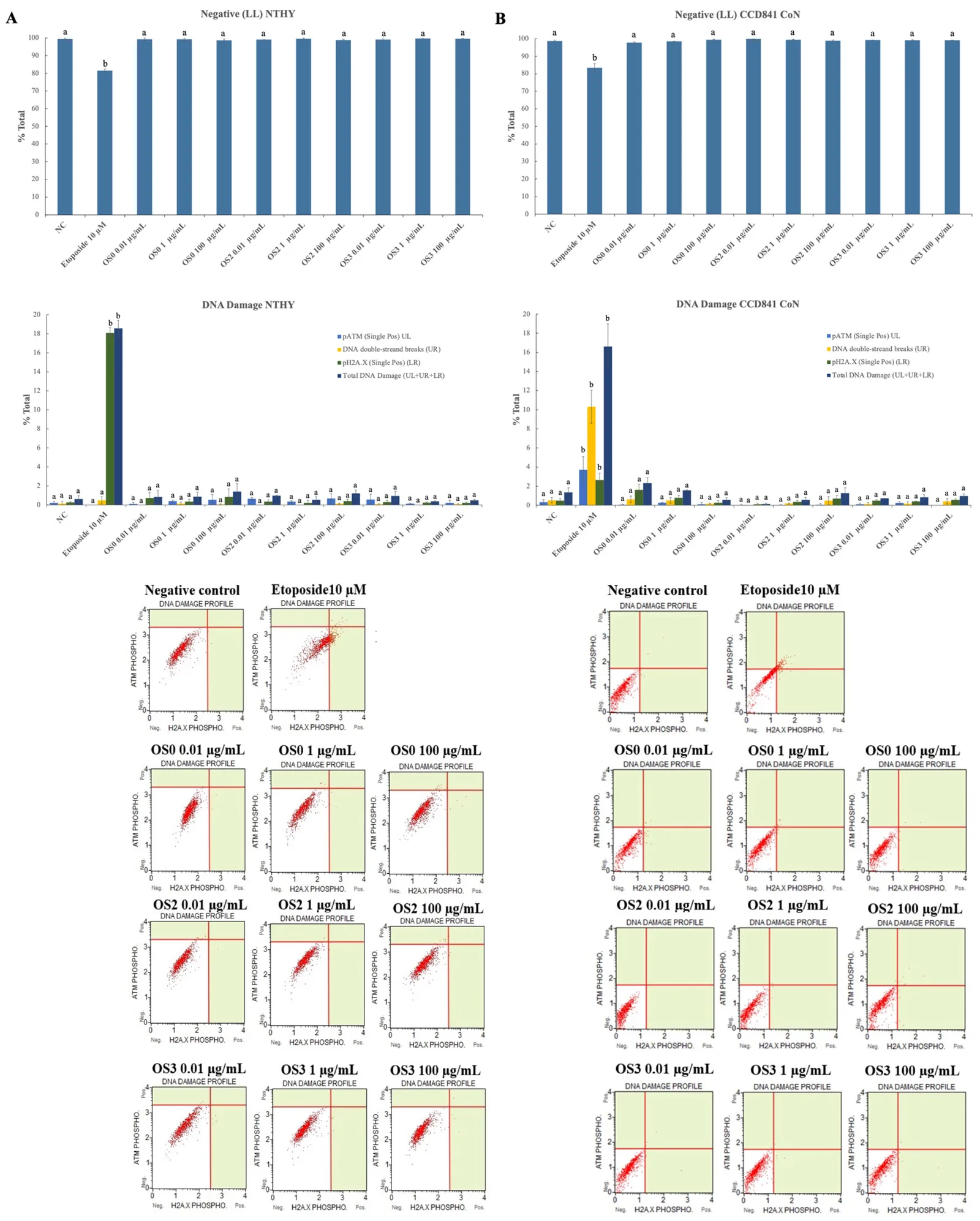
DNA damage marker profiles (γH2AX, DSBs, pATM) in normal human thyroid epithelial cells Nthy-ori 3-1 (NTHY) (**A**) and normal human colonic epithelial cells (CCD 841 CoN) (**B**) following exposure to chitosan-alginate film digestates at concentrations of 0.01, 1, and 100 µg/mL. Data represent mean ± SD (n = 3). Values followed by the same letters are not significantly different at *p* < 0.05 (one-way ANOVA with Tukey post hoc test). Untreated controls (NC), Etoposide—positive control (10 µM), and digestates from OS0, OS2, and OS3 films.

DNA damage assessment revealed that digestates derived from chitosan-alginate films enriched with turmeric and oregano two-phase equilibrium condensates (OS2 and OS3) did not induce detectable DNA damage in either Nthy-ori 3-1 thyroid epithelial cells or CCD 841 CoN colonic epithelial cells (Figure 5A,B). In contrast, etoposide induced clear DNA-damage responses, confirming the responsiveness of the assay.

Untreated control cells (NC) and cells exposed to base chitosan-alginate film digestate (OS0) showed low background levels of the evaluated markers (Figure 5A,B). In Nthy-ori 3-1 cells, pATM, DSBs, γH2AX, and total DNA damage were 0.20 ± 0.17%, 0.13 ± 0.23%, 0.27 ± 0.06%, and 0.60 ± 0.36%, respectively (Figure 5A). Corresponding values in CCD 841 CoN cells were 0.32 ± 0.28%, 0.53 ± 0.28%, 0.49 ± 0.25%, and 1.34 ± 0.50%, respectively (Figure 5B).

Etoposide, used as the positive control, induced pronounced DNA-damage responses in both cell lines (Figure 5A,B). In Nthy-ori 3-1 cells, γH2AX increased approximately 67-fold relative to the untreated control (Figure 5A). In CCD 841 CoN cells, etoposide increased pATM to 3.73 ± 1.38%, DSBs to 10.30 ± 1.73%, γH2AX to 2.63 ± 0.75%, and total DNA damage to 16.62 ± 2.35% (Figure 5B).

In contrast to etoposide, OS2 and OS3 digestates (0.01–100 µg/mL) did not significantly alter the evaluated DNA-damage markers in either Nthy-ori 3-1 or CCD 841 CoN cells (all *p* > 0.05 vs. NC) (Figure 5A,B). This finding is consistent with previous reports describing limited cytotoxic or genotoxic effects of chitosan-based systems in non-malignant cell models at comparable concentrations [31]. Although carvacrol and thymol, the major bioactive constituents associated with oregano, have been reported to reduce irradiation-associated DNA damage in non-malignant tissues [32], the composition and release of individual constituents from the OS3 digestate were not determined in the present study. Similarly, chitosan-based formulations containing ferulic acid or curcumin have been reported to modulate DNA-damage signaling in cancer-cell models while showing limited effects in non-malignant cells [33,34]. However, direct comparison with these systems should be made cautiously because of differences in formulation, cell model, and exposure conditions.

Overall, the absence of significant changes in pATM, DSBs, γH2AX, or total DNA damage indicates that the tested OS2 and OS3 film digestates did not elicit a detectable DNA-damage response in the evaluated epithelial cell lines under the applied experimental conditions. This finding supports a favorable short-term in vitro genotoxicity profile, but it does not establish comprehensive safety. In particular, the assessment was limited to acute exposure in two selected cell lines and did not address chronic or repeated exposure. Moreover, the concentration of individual constituents migrating from the films into blueberries was not measured. The cell-cycle alterations observed in Nthy-ori 3-1 cells following exposure to OS2 and OS3 also indicate biological activity, although they were not accompanied by significant increases in the evaluated DNA-damage markers and therefore cannot, by themselves, be interpreted as evidence of genotoxicity.

## 3. Materials and Methods

### 3.1. Bioactive Condensate Extraction

Dried oregano leaves were first pulverized using an electric herb grinder operating at 26,000 r/min. Turmeric powder was procured from a local bio supermarket. Subsequently, 20 g of either oregano or turmeric powder was mixed with 200 mL of sterilized water in a 500 mL volumetric flask. Hydrodistillation was performed for 2 h using a Clevenger-type apparatus. After condensation, the distillate separated into two immiscible phases: an oil-rich phase (essential oil) and an aqueous phase (hydrosol/hydrodistillate). Both phases were collected and stored at 4 °C in amber containers. Prior to incorporation into the film-forming matrix, the two phases were recombined by vigorous shaking to obtain a biphasic dispersion; because the phases are immiscible, samples were used immediately after shaking and were shaken again before each dosing/aliquot withdrawal.

These condensates were characterized in our previous work, revealing TPC values of 3.14 ± 0.12 µmol GAE/g for turmeric and 78.23 ± 5.76 µmol GAE/g for oregano, confirming their differing antioxidant potentials [5].

### 3.2. Chitosan-Alginate Film Preparation and Characterization

#### 3.2.1. Film Preparation

The preparation of the active chitosan-alginate films followed our previously described protocol [5], with adjustments made solely to the drying phase. Initially, a 1% (*w*/*v*) chitosan dispersion (medium molecular weight; Sigma-Aldrich, St. Louis, MO, USA) in 1% (*v*/*v*) acetic acid was filtered at ambient temperature, while a 2% (*w*/*v*) sodium alginate solution (Sigma-Aldrich, St. Louis, MO, USA) was dissolved in sterile water under continuous agitation at 70 °C. The biopolymers were blended at a constant chitosan-to-alginate mass ratio of 1:0.25 (*w*/*w*) and plasticized with 1% (*v*/*v*) glycerol (StanLab, Lublin, Poland), then stirred for 30 min at room temperature before undergoing high-shear homogenization (15,000 rpm for 5 min) to ensure structural uniformity. While the control formulation (OS0) remained unsupplemented, the active films were functionalized with two-phase equilibrium condensates: turmeric was integrated to reach a chitosan:alginate:turmeric mass ratio of 1:0.25:2 (*w*/*w*) for the OS2 matrices, and oregano was added at a 1:0.25:1 (*w*/*w*) ratio for the OS3 variants. These specific inclusion levels were established during our earlier optimization of equivalent systems, wherein they exhibited significant antimicrobial efficacy against foodborne pathogens without compromising biocompatibility in human cellular models [5].

Following solution homogenization, the prepared film-forming solutions were cast into sterile Petri dishes and dried under a laboratory fume hood at room temperature (22 ± 2 °C) for 48 h. This process resulted in the formation of flexible films with uniform thickness, which were subsequently peeled from the casting surfaces prior to characterization and application as food packaging.

The physicochemical, mechanical, and thermal properties of the chitosan-alginate films (OS0, OS2, OS3) prepared in this study were comprehensively characterized in our previous publication [5]. Key findings included ATR-FTIR confirmation of chitosan-alginate ionic interactions (1427 cm^−1^ carboxylate, 1590 cm^−1^ amide), UV-Vis peaks at 245 nm (OS2-turmeric) and 225/275 nm (OS3-oregano) verifying bioactive incorporation, enhanced thermal stability (↑10 °C by TGA), and improved mechanical performance in OS3 (40% higher tensile strength versus OS0) [5].

#### 3.2.2. Visual Appearance and Homogeneity of the Films

Following the drying protocol, the formulated chitosan-alginate films (OS0, OS2, and OS3) were cut into standardized 5 × 5 cm specimens to assess their visual appearance and structural homogeneity. The macroscopic evaluation was conducted under standardized laboratory illumination to determine structural continuity, including the presence or absence of cracks, pinholes, or phase separation. Additionally, the overall coloration and optical profile of the polymeric matrices were qualitatively recorded, and structural homogeneity was confirmed through optical magnification (10×) to ensure the absence of particulate aggregates or phase separation exceeding 0.1 mm. All macroscopic inspections were performed in triplicate (n = 3 independent sheets per formulation) prior to their application in active packaging or in vitro digestion assays.

#### 3.2.3. Water Absorption and Swelling Analysis of Films

Film samples (1.5 cm × 1.5 cm) were subjected to gravimetric water absorption and swelling measurements according to established protocols. Initial sample mass ($M_{\mathrm{initial}}$) was recorded following desiccation in a desiccator for 24 h containing anhydrous calcium chloride at 0% relative humidity. Samples were weighed periodically until the difference between consecutive measurements was less than 0.001 g, confirming that practical weight equilibrium had been reached. Samples were then oven-dried at 105 ± 2 °C for 24 h to determine dry mass ($M_{\mathrm{dry}}$). Following equilibration in a desiccator to constant mass, film samples were immersed in 20 mL of distilled water at room temperature (22 ± 2 °C) for 24 h. After immersion, excess surface moisture was carefully blotted using filter paper applied for no more than 5 s per side to standardize surface water removal, and swollen mass ($M_{\mathrm{swollen}}$) was immediately recorded to minimize water evaporation. All measurements were performed in quintuplicate (n = 5) per formulation.

Three parameters characterizing film hydration behavior were calculated using standard gravimetric formulas. Moisture content (MC, %) was determined using the formula:

$$\mathrm{MC}=\frac{M_{\mathrm{initial}}-M_{\mathrm{dry}}}{M_{\mathrm{initial}}}\times 100(\%)$$

which expresses the water content relative to the initial sample mass.

Water uptake (WU, %) was calculated as:

$$\mathrm{WU}=\frac{M_{\mathrm{swollen}}-M_{\mathrm{dry}}}{M_{\mathrm{dry}}}\times 100(\%)$$

quantifying the amount of water absorbed relative to the dry polymer mass, thereby reflecting the hydration capacity of the polymer network.

Swelling degree (SD, %), expressed as:

$$\mathrm{SD}=\frac{M_{\mathrm{swollen}}-M_{\mathrm{initial}}}{M_{\mathrm{initial}}}\times 100(\%)$$

represents the dimensional expansion of the film upon water absorption, indicating the extent of polymer chain relaxation and free volume increase within the three-dimensional network. These three parameters collectively provide comprehensive characterization of film hydration kinetics and dimensional stability, assessing the performance of composite materials in aqueous environments.

### 3.3. Blueberry Sample Preparation and Packaging Configuration

Fresh blueberries (*Vaccinium corymbosum* L.) were procured from a local commercial supplier (Kraków, Poland) and selected based on uniform ripeness, absence of visible defects, and consistent size distribution. The fruits were randomly distributed, meticulously weighed to 100 ± 2 g, and assigned to different packaging systems. Blueberries were packed either in commercial plastic retail clamshell containers (Cp; rigid transparent PET, acting as a reference) or in active pouches formed from free-standing chitosan-alginate films (OS0, OS2, and OS3; flexible sheets, thickness ~250 µm). To evaluate the practical postharvest efficacy of the active films in a laboratory-scale proof-of-concept configuration, the polymeric sheets were converted into uniform rectangular pouches measuring 15 cm × 12 cm, corresponding to a total film surface area of approximately 360 cm^2^. Because the cross-linked chitosan-alginate matrices lack intrinsic thermo-sealing properties, the structural integrity of the pouches was maintained using mechanical clips. To facilitate adequate atmospheric gas exchange for the respiring fruit, similar to the ventilation present in conventional clamshell packaging, and to prevent excessive internal condensation, a standardized macro-perforation geometry was applied. Specifically, 12 evenly distributed linear incisions (approximately 1 cm in length each) were made across the film surface using a sterile surgical scalpel prior to packing. This configuration yielded a film surface-area-to-product-mass ratio of approximately 3.6 cm^2^/g, deliberately chosen to ensure sufficient headspace saturation with the released bioactive volatiles.

### 3.4. Storage Conditions

Following packaging, the blueberries were stored under two distinct environmental regimes to evaluate temperature-dependent changes in quality attributes and microbial load. The cold storage at 4 °C was conducted in a temperature-controlled room with a high relative humidity (85 ± 5% RH), mimicking commercial refrigerated fruit storage. In contrast, the ambient storage at 22 °C was carried out under natural laboratory humidity (50 ± 10% RH), simulating non-refrigerated retail or household “thermal abuse” scenarios. These conditions do not strictly replicate all postharvest commercial scenarios (e.g., retail display at intermediate ‘abuse’ temperatures such as 8–10 °C) but rather provide a controlled comparison of thermal effects and their interactions with packaging formulation.

Following each storage period, samples were partitioned into two groups: fresh material for immediate analysis and freeze-dried material. For lyophilization, blueberries were initially frozen at −80 °C for 24 h to ensure complete ice crystal formation and optimal structural preservation. Subsequently, samples were processed using a Christ Alpha 1-4 freeze dryer (Martin Christ Gefriertrocknungsanlagen GmbH, Osterode am Harz, Germany) operating under standard conditions. The freeze-drying cycle was maintained until moisture content reached equilibrium. Lyophilized samples were subsequently pulverized using a laboratory mill (FRITSCH Pulverisette 14; FRITSCH GmbH, Idar-Oberstein, Germany) to achieve uniform particle size distribution and enhance extraction efficiency. Ground samples were transferred to sealed polyethylene bags and maintained at −20 °C until further analysis.

### 3.5. Blueberry Analyses

#### 3.5.1. Determination of Dry Matter Content and Basic Chemical Composition

The dry matter content of the analyzed blueberries (*Vaccinium corymbosum* L.) was determined by the drying oven method according to Polish Standard PN-A-79011-3:1998 [35]. This method is based on mass loss resulting from water evaporation during thermal drying under atmospheric pressure. The dry matter content was expressed as a percentage of the initial sample weight.

The basic chemical composition of freeze-dried blueberry samples, including ash, crude fat, total dietary fiber, and protein, was analyzed following the respective Polish Standards (PN).

Ash content was determined by dry ashing in a muffle furnace at 525 ± 25 °C following PN-A-79011-8:1998 [36].

Crude fat was extracted by the Soxhlet method (PN-A-79011-4:1998) [37] using a Soxtec Avanti 2050 Auto System (Foss Tecator AB, Höganäs, Sweden).

Total dietary fiber was determined enzymatically using the Total Dietary Fiber Assay Kit (Megazyme, Sydney, Australia) in accordance with PN-A-79011-15:1998 [38].

Total nitrogen was determined by the Kjeldahl method (ISO 8968-1:2004) [39] and converted to crude protein using a nitrogen-to-protein conversion factor of 6.25. The analysis was performed using a Kjeltec 2200 distillation system (Foss Tecator AB, Höganäs, Sweden).

#### 3.5.2. Determination of Phenolic Compounds and Antioxidant Capacity

##### Extraction Procedure

Freeze-dried blueberry samples were extracted with 70% (*v*/*v*) methanol acidified with 0.1% formic acid and used for the determination of total phenolic content (TPC) and antioxidant activity (ABTS^·+^ and DPPH assays).

##### Total Phenolic Content (Folin–Ciocalteu Method)

Total phenolic content (TPC) was quantified spectrophotometrically using the Folin–Ciocalteu method following Swain and Hillis [40]. Absorbance was measured at 760 nm against a blank containing 0.1% formic acid in 70% methanol, and the results were expressed as chlorogenic acid equivalents per 100 g^−1^ dry weight.

##### ABTS Radical Cation Scavenging Capacity

The antioxidant capacity of blueberry extracts was assessed using the ABTS^·+^ radical cation decolorization assay according to the method described by Re et al. [41]. Briefly, the ABTS^·+^ solution was prepared by reacting 3.84 mg of crystalline ABTS (2,2′-azino-bis(3-ethylbenzothiazoline-6-sulfonic acid)) with 66.2 mg of potassium persulfate (K_2_S_2_O_8_) in 10 mL of distilled water, and incubating the mixture in the dark at room temperature for 16 h. The working solution was then diluted with distilled water to obtain an absorbance of 0.740–0.770 at 734 nm.

An aliquot of the methanolic blueberry extract was mixed with the ABTS^·+^ working solution, incubated at room temperature for 6 min, and the absorbance was measured at 734 nm against a blank. The percentage of radical scavenging activity (RSA) was calculated according to the formula:

$$\mathrm{RSA}=\frac{E_{1}-E_{2}}{E_{1}}\times 100$$

where $E_{1}$ is the absorbance of the ABTS^·+^ solution after dilution, and $E_{2}\;$ is the absorbance measured 6 min after sample addition.

The decrease in absorbance of the blue-green ABTS^·+^ solution was proportional to the antioxidant capacity of the extracts. Antioxidant capacity was quantified using a Trolox calibration curve and expressed as μmol Trolox equivalents per g dry weight (μmol TE g^−1^ dw).

##### DPPH Radical Scavenging Capacity

The antioxidant capacity of blueberry extracts was assessed by the DPPH (2,2-diphenyl-1-picrylhydrazyl) radical assay, following the protocol of Miliauskas et al. [42]. Briefly, a solution of DPPH radicals was prepared by dissolving 5 mg of crystalline DPPH in 100 mL of methanol. Sample extracts were mixed with DPPH solution and incubated for 10 min at room temperature. Absorbance was measured spectrophotometrically at 515 nm, and the percent radical scavenging activity (%RSA) was calculated using the formula:

$$\mathrm{RSA}=\frac{A_{\mathrm{blank}}-A_{\mathrm{sample}}}{A_{\mathrm{blank}}}\times 100$$

where $A_{\mathrm{blank}}$ is the absorbance of the DPPH solution without sample, and $A_{\mathrm{sample}}$ is the absorbance in the presence of the blueberry extract. A standard curve was constructed using Trolox solutions and results were expressed as Trolox equivalents (TE) per gram of dry weight.

#### 3.5.3. Determination of Vitamin C Content (L-Ascorbic Acid)

Blueberry homogenate (2.00 ± 0.01 g) was quantitatively transferred to a 100 mL volumetric flask containing 80 mL metaphosphoric acid (20 g L^−1^). The suspension was vigorously shaken, adjusted to volume, and filtered.

Filtered extract (20 mL) was combined with L-cysteine hydrochloride (10 mL, 40 g L^−1^) and stirred magnetically for 5 min. The pH was sequentially adjusted to 7.0–7.2 using sodium phosphate buffer (200 g L^−1^, 5 min stirring), followed by acidification to pH 2.5–2.8 with metaphosphoric acid (200 g L^−1^). The reduced extract was quantitatively transferred to a 50 mL volumetric flask, brought to volume with ultrapure water, and filtered prior to analysis.

L-Ascorbic acid standards (1–100 µg mL^−1^) were prepared daily in metaphosphoric acid (20 g L^−1^). Total vitamin C (L-ascorbic acid + reduced dehydro-L-ascorbic acid) was quantified using a C18 column (250 × 4.6 mm, 5 µm) under isocratic elution with 0.01% acetic acid and methanol (90:10, *v*/*v*) (0.7 mL min^−1^) and UV detection at 254 nm (20 µL injection). Results were calculated from peak areas against the external standard curve (R^2^ ≥ 0.999) and expressed as mg 100 g^−1^ fresh weight. Determinations were performed in triplicate.

#### 3.5.4. Determination of Total Viable Bacterial Count (TVC) in Blueberries Stored in Active Films

The total viable bacterial count (TVC), as a quantitative estimate of viable microorganisms, was determined according to ISO 4833-1:2013 [43]. Results were expressed as log colony-forming units per gram (log CFU/g) of fresh blueberry tissue after incubation on Plate Count Agar (PCA; Cat. No. CM0325, Oxoid, Thermo Fisher Scientific, Basingstoke, UK) at 30 °C for 72 h.

This analysis was included to evaluate microbial contamination levels, which directly determine the hygienic quality and shelf-life of the stored blueberries. The antimicrobial efficacy of chitosan-alginate films enriched with turmeric and oregano two-phase equilibrium condensates was quantified by comparing TVC values in fruit packaged in bioactive films (OS2, OS3) versus control non-bioactive films (OS0) across the three storage regimes (4 °C for 7 days, 22 °C for 7 days, and 4 °C for 14 days). Blueberries stored in conventional macro-perforated rigid PET clamshells served as an additional control (C_p_) to benchmark the antimicrobial performance of the novel chitosan-alginate films against industry-standard packaging materials.

### 3.6. In Vitro Digestion of the Films

Film samples (0.50 ± 0.01 g) underwent sequential three-stage gastrointestinal digestion following the standardized static in vitro model [44]. Simulated salivary fluid (SSF, pH 7.0), gastric fluid (SGF, pH 3.0), and intestinal fluid (SIF, pH 7.0) were prepared with physiological electrolytes. The oral phase (2 min, 37 °C) employed human salivary α-amylase (75 U mL^−1^). The gastric phase (120 min, 37 °C) utilized porcine pepsin (2000 U mL^−1^) following pH adjustment to 3.0 with HCl. The intestinal phase (120 min, 37 °C) incorporated porcine pancreatin (100 U mL^−1^ trypsin activity) and bile salts (10 mM) after neutralization to pH 7.0 with NaOH. The obtained post-digestion supernatants were subsequently collected and used in the intestinal absorption model.

### 3.7. Caco-2 Barrier Integrity Verification and Basolateral Sampling

Caco-2 cells (human colorectal adenocarcinoma, Sigma-Aldrich, 86010202, St. Louis, MO, USA) were chosen as the gold standard model for intestinal epithelium due to their ability to differentiate into enterocyte-like cells with characteristic brush border morphology and functional transport properties. Upon confluency and differentiation (21 days), Caco-2 monolayers effectively simulate human intestinal absorption, making them ideal for assessing the safety of compounds that may migrate from food packaging materials. Cells were cultured in Minimum Essential Medium Eagle (MEM; Sigma-Aldrich, M7145, St. Louis, MO, USA), supplemented with 2 mM L-glutamine (Sigma-Aldrich, G7513, St. Louis, MO, USA), 1% MEM Non-Essential Amino Acid Solution (100×; Sigma-Aldrich, M7145, St. Louis, MO, USA), 10% fetal bovine serum (ATCC^®^ 30-2020™, Manassas, VA, USA), and antibiotic solution containing penicillin (10,000 U mL^−1^) and streptomycin (10,000 µg mL^−1^) (Sigma-Aldrich, P0781, St. Louis, MO, USA), added at a final concentration of 10 mL L^−1^.

Caco-2 cells (5 × 10^4^ cells/well) were seeded into apical compartments of 12-well 0.4 μm PET Transwell inserts (Greiner Bio-One, Kremsmünster, Austria) with 1 mL culture medium added to both apical and basolateral compartments. Medium was replaced every 2–3 days. After 21 days of culture, Caco-2 monolayers exhibited intestinal enterocyte morphology and physiology. Monolayer integrity was verified by transepithelial electrical resistance (TEER) measurements using an EVOM electrode (World Precision Instruments, Sarasota, FL, USA), with values > 500 Ω·cm^2^, confirming robust barrier function prior to basolateral fraction collection. Digested films were applied to the apical surface of Caco-2 monolayers and incubated for 2 h. Basolateral compartment filtrates were collected for subsequent analysis.

### 3.8. Cell Lines and Culture Conditions

Two distinct cell lines were selected to evaluate the comprehensive safety profile of chitosan-alginate films enriched with oregano or turmeric condensates, representing key target tissues for food packaging applications and potential exposure pathways.

Nthy-ori 3-1 cells (human thyroid follicular epithelial cells, Sigma-Aldrich, 90011609, St. Louis, MO, USA) were selected to assess potential endocrine disruption effects, as the thyroid gland is particularly sensitive to xenobiotic compounds and represents an important target organ for food safety evaluation. These immortalized normal thyroid cells maintain physiological characteristics of follicular epithelium while providing a reproducible model for endocrine toxicity screening. Cells were maintained in RPMI-1640 Medium (Sigma-Aldrich, R8758, St. Louis, MO, USA), supplemented with 2 mM L-glutamine (Sigma-Aldrich, G7513, St. Louis, MO, USA), 10% fetal bovine serum (ATCC^®^ 30-2020™, Manassas, VA, USA), and antibiotic solution containing penicillin (10,000 U mL^−1^) and streptomycin (10,000 µg mL^−1^) (Sigma-Aldrich, P0781, St. Louis, MO, USA), added at 10 mL L^−1^.

CCD 841 CoN cells (human normal colon epithelial cells, ATCC^®^ CRL-1790™, Manassas, VA, USA) served as a normal, non-transformed control model representing healthy colon epithelium. CCD 841 CoN cells were cultured in Eagle’s Minimum Essential Medium (EMEM; ATCC^®^ 30-2003™, Manassas, VA, USA), supplemented with 10% fetal bovine serum (ATCC^®^ 30-2020™, Manassas, VA, USA) and Penicillin-Streptomycin Solution containing penicillin (10,000 U mL^−1^) and streptomycin (10,000 µg mL^−1^) (ATCC^®^ 30-2300™, Manassas, VA, USA).

Cell lines were maintained at 37 °C in a humidified atmosphere containing 5% CO_2_ and 95% air using a CO_2_ incubator (NuAire, Plymouth, MN, USA).

Nthy-ori 3-1 and CCD 841 CoN cells were seeded at 2 × 10^5^ cells/well in 6-well plates containing 2 mL complete growth medium and incubated for 24 h to establish exponential growth phase. Medium was then replaced with fresh growth medium containing film digestate filtrates at concentrations of 0.01, 1, and 100 μg mL^−1^ (scaled to represent realistic physiological exposure based on human intestinal surface area migration estimates). Cells cultured in growth medium alone served as negative controls (NC). For cell cycle analysis, staurosporine (1.5 μM; Sigma-Aldrich, St. Louis, MO, USA) was used as a positive control. DNA damage assessment employed etoposide (10 μM) as a positive control. All treatments were conducted for 24 h at 37 °C in 5% CO_2_.

### 3.9. Cell Cycle Analysis

Cell cycle phase distribution and proliferative status of treated cells were evaluated using the Muse Cell Cycle assay (Cytek Biosciences, Fremont, CA, USA), which detects DNA content via propidium iodide (PI) staining to assess progression through distinct phases of the cell cycle. The assay employs single-color flow cytometry analysis to identify three distinct cell populations based on DNA content: G0/G1-phase cells, S-phase cells, and G2/M-phase cells. Staurosporine (1.5 μM; Sigma-Aldrich, St. Louis, MO, USA), a broad-spectrum kinase inhibitor known to induce potent cell cycle arrest and apoptosis, served as the positive control. Untreated cells in complete growth medium served as the negative control (NC). Following 24 h exposure, cells were harvested by trypsinization, resuspended in complete growth medium, and processed for the Muse Cell Cycle assay according to the manufacturer instructions. All measurements were performed in triplicate (n = 3).

### 3.10. DNA Damage Assessment

Genotoxic potential of film digestate filtrates was evaluated using the Muse Multi-Color DNA Damage assay (Cytek Biosciences, Fremont, CA, USA), which simultaneously detects phosphorylated ATM (pATM) at Ser1981 and phosphorylated histone H2A.X (pH2A.X) at Ser139 to assess DNA damage response activation and double-strand breaks. Etoposide (10 μM) and untreated cells served as positive and negative controls, respectively. Following fixation, permeabilization, and staining with antibodies against pATM and pH2A.X, flow cytometric analysis was performed according to the manufacturer instructions, and results were expressed as a percentage of cells in four populations: undamaged, pATM single positive, pH2A.X single positive, and dual positive (active double-strand breaks). All measurements were performed in triplicate (n = 3).

### 3.11. Statistical Analysis

For the blueberry storage experiment, three separate packages were prepared for each combination of film formulation, storage temperature, and storage time. The films used to prepare the packages were cast from the same batch of film-forming solution. Each package was treated as one experimental replicate (n = 3). Randomly selected blueberries were sampled separately from each package, with the sample mass adjusted to the requirements of the respective physicochemical and microbiological analysis. Depending on the analytical method, one measurement or technical replicates were performed for each sample collected from an individual package. Where technical replicates were performed, their mean value was used for statistical analysis. Thus, physicochemical and microbiological data were analyzed using three package-level experimental replicates, and technical replicates were not treated as independent observations.

Cell-based experiments were performed in at least three independent experiments, each including at least three technical replicates per treatment condition. For each independent experiment, cells were exposed to the respective film digestate concentration or control under the same experimental conditions. The mean value of the technical replicates was used for statistical analysis, and technical replicates were not treated as independent observations.

Quantitative data were expressed as mean ± standard deviation (SD). The normality of data distribution was assessed using the Shapiro–Wilk test. One-way ANOVA was used for single-factor comparisons, whereas multifactorial ANOVA was applied to assess the main effects and interactions of packaging type, storage temperature, and storage time. When significant effects were detected (*p* < 0.05), Tukey’s honestly significant difference (HSD) post hoc test was used for multiple pairwise comparisons. Statistical significance was set at *p* < 0.05. All analyses were performed using Statistica 13.3 PL software (StatSoft, Inc., Tulsa, OK, USA).

## 4. Conclusions

This study demonstrates the laboratory-scale, proof-of-concept development of chitosan-alginate films enriched with oregano or turmeric biphasic condensates. The results highlight distinct physicochemical behaviors of the active matrices: oregano-enriched films (OS3) exhibited significantly greater water uptake and swelling than the control and turmeric-enriched films, whereas OS2 showed hydration behavior comparable to that of the control matrix. These results indicate formulation-dependent differences in the physical behavior of the films. Although the greater swelling of OS3 may conceptually support moisture-triggered release of bioactive volatiles in macro-perforated packaging, active-compound release, film microstructure, and migration into blueberries or package headspace were not directly measured.

During the preliminary blueberry storage trials, blueberries packaged with OS2 and OS3 films showed higher vitamin C contents than those in conventional plastic packaging after 14 days of storage at 4 °C. This finding was specific to the tested refrigerated storage condition and was not observed consistently across all treatments and storage conditions. OS3 was associated with higher antioxidant capacity under ambient-temperature storage. These findings should be interpreted as formulation- and condition-specific effects within the tested blueberry storage model rather than as evidence of general preservation superiority. Importantly, microbiological analysis showed that total viable bacterial counts were predominantly influenced by storage temperature, whereas packaging type did not demonstrate a significant main effect. Therefore, the present findings do not demonstrate antimicrobial efficacy of the active films, and these materials cannot be considered a substitute for strict cold-chain management.

Initial in vitro safety screening showed that OS2 and OS3 film digestates did not significantly increase the evaluated DNA-damage markers in Nthy-ori 3-1 and CCD 841 CoN cells under the applied conditions. This supports a favorable short-term in vitro genotoxicity profile of the tested digestates, rather than comprehensive safety. The conclusion is limited by the short exposure period, the use of two selected cell lines, and the endpoints assessed. In addition, the cell-cycle changes observed in Nthy-ori 3-1 cells after exposure to OS2 and OS3 indicate biological activity, although they were not accompanied by increased DNA-damage markers and cannot alone be interpreted as evidence of genotoxicity. These short-term results therefore cannot be generalized to long-term human safety or chronic in vivo toxicity.

To avoid premature conclusions regarding commercial viability and safety, further investigations are required. The present conclusions are limited to the tested formulations, the laboratory-scale blueberry storage set-up, and the measured endpoints. Future research should include: (i) microstructural and morphological evaluation, including scanning electron microscopy, to characterize the polymer matrix before and after hydration; (ii) measurements of active-compound migration into blueberries, release kinetics into package headspace, and their possible effects on fruit respiration, including O_2_, CO_2_, and ethylene dynamics; (iii) sensory evaluation to determine possible effects on fruit aroma, flavor, appearance, and consumer acceptance; (iv) expanded microbiological evaluation, including yeast and mold enumeration and targeted challenge tests against relevant postharvest pathogens, such as *Botrytis cinerea*; (v) long-term mechanical-performance and functional-stability testing during storage, handling, and potential scale-up; and (vi) advanced toxicological profiling, including repeated-exposure and long-term in vivo studies. Finally, given the moisture sensitivity of these hydrophilic matrices, future industrial development should assess whether their use as moisture-activated inserts or active inner coatings on conventional thermo-sealable substrates is more feasible than their application as free-standing bags.

## Figures and Tables

**Table 1:** Effect of packaging type, storage temperature, and time on the dry matter content and basic chemical composition of highbush blueberry (*Vaccinium corymbosum* L.).

Sample	Dry Matter [% Dry Weight]	Protein [% Dry Weight]	Crude Fat [g/100 g Dry Weight]	Dietary Fiber [g/100 g Dry Weight]	Ash [%/100 g Dry Weight]
C	12.13 ^a^ ± 1.22	2.88 ^a^ ± 0.24	0.82 ^def^ ± 0.004	8.82 ^a^ ± 1.04	1.09 ^a^ ± 0.08
Day 7 4 °C					
Cp	12.55 ^ab^ ± 0.72	2.80 ^a^ ± 0.16	0.89 ^ef^ ± 0.02	9.68 ^a^ ± 0.52	1.12 ^a^ ± 0.05
OS0	12.60 ^ab^ ± 1.91	2.65 ^a^ ± 0.001	0.73 ^abcd^ ± 0.07	12.14 ^a^ ± 3.29	1.13 ^a^ ± 0.15
OS2	13.29 ^ab^ ± 3.19	2.72 ^a^ ± 0.02	0.91 ^f^ ± 0.01	13.07 ^a^ ± 2.99	1.23 ^a^ ± 0.02
OS3	12.41 ^ab^ ± 1.65	2.73 ^a^ ± 0.21	0.87 ^ef^ ± 0.05	9.07 ^a^ ± 0.54	1.04 ^a^ ± 0.09
Day 7 22 °C					
Cp	13.10 ^ab^ ± 0.92	3.09 ^a^ ± 0.25	0.78 ^cde^ ± 0.07	14.72 ^a^ ± 1.96	1.09 ^a^ ± 0.08
OS0	16.69 ^b^ ± 0.74	2.71 ^a^ ± 0.02	0.77 ^cde^ ± 0.0003	13.67 ^a^ ± 3.34	1.11 ^a^ ± 0.21
OS2	13.93 ^ab^ ± 1.32	2.75 ^a^ ± 0.03	0.94 ^f^ ± 0.0004	12.68 ^a^ ± 3.20	1.32 ^a^ ± 0.007
OS3	13.45 ^ab^ ± 1.35	3.12 ^a^ ± 0.02	0.70 ^abcd^ ± 0.03	10.49 ^a^ ± 6.16	1.21 ^a^ ± 0.01
Day 14 4 °C					
Cp	9.99 ^a^ ± 1.41	2.84 ^a^ ± 0.11	0.81 ^def^ ± 0.06	12.62 ^a^ ± 3.92	1.25 ^a^ ± 0.06
OS0	11.49 ^a^ ± 1.21	2.67 ^a^ ± 0.34	0.59 ^a^ ± 0.06	8.54 ^a^ ± 0.56	1.04 ^a^ ± 0.11
OS2	12.81 ^ab^ ± 1.15	2.77 ^a^ ± 0.15	0.61 ^ab^ ± 0.02	13.67 ^a^ ± 1.87	1.31 ^a^ ± 0.03
OS3	11.79 ^a^ ± 1.51	2.80 ^a^ ± 0.33	0.67 ^abc^ ± 0.07	11.97 ^a^ ± 5.29	1.15 ^a^ ± 0.23

Data are presented as mean ± SD of three package-level experimental replicates (n = 3). Different superscript letters within the same column indicate statistically significant differences among treatment combinations at *p* < 0.05, as determined by multifactorial ANOVA followed by Tukey’s post hoc test. C—control sample, day 0; Cp—standard macro-perforated plastic packaging; OS0—chitosan:alginate, 1:0.25 (*w*/*w*) film; OS2—chitosan:alginate:turmeric, 1:0.25:2 (*w*/*w*) film, OS3—chitosan:alginate:oregano, 1:0.25:1 (*w*/*w*) film.

**Table 2:** Effect of packaging type, storage temperature, and time on antioxidant activity (ABTS, DPPH), total phenolic content (TPC), and vitamin C content in highbush blueberry (*Vaccinium corymbosum* L.).

Sample	TPC [mg Chlorogenic Acid Equivalents/100 g Dry Weight]	ABTS Activity [μmol Trolox/g Dry Weight]	DPPH Activity [μmol Trolox/g Dry Weight]	Vitamin C [mg/100 g Fresh Weight]
C	1.21 ^a^ ± 0.07	188.56 ^abcde^ ± 6.08	93.53 ^bcde^ ± 6.45	25.71 ^i^ ± 0.44
Day 7 4 °C				
Cp	1.32 ^a^ ± 0.13	160.44 ^a^ ± 7.96	93.00 ^bcde^ ± 6.57	14.16 ^f^ ± 0.21
OS0	1.45 ^ab^ ± 0.10	175.31 ^ab^ ± 28.29	82.95 ^ab^ ± 14.36	13.61 ^de^ ± 0.20
OS2	1.35 ^ab^ ± 0.02	178.67 ^abcd^ ± 5.81	83.35 ^abc^ ± 6.87	14.94 ^g^ ± 0.22
OS3	1.74 ^abc^ ± 0.48	187.51 ^abcde^ ± 29.98	96.42 ^bcde^ ± 9.51	15.89 ^h^ ± 0.08
Day 7 22 °C				
Cp	1.96 ^bc^ ± 0.40	210.43 ^def^ ± 21.90	99.96 ^cde^ ± 10.85	14.29 ^f^ ± 0.50
OS0	1.60 ^abc^ ± 0.03	197.57 ^abcde^ ± 10.63	100.55 ^de^ ± 0.94	14.08 ^ef^ ± 0.13
OS2	1.89 ^abc^ ± 0.14	213.10 ^ef^ ± 12.07	106.28 ^e^ ± 1.57	12.93 ^c^ ± 0.30
OS3	2.30 ^c^ ± 0.09	237.40 ^f^ ± 12.81	89.17 ^abcd^ ± 3.10	15.35 ^g^ ± 0.24
Day 14 4 °C				
Cp	1.32 ^a^ ± 0.13	173.94 ^abc^ ± 31.76	83.09 ^ab^ ± 11.29	11.44 ^a^ ± 0.27
OS0	1.48 ^ab^ ± 0.39	172.25 ^ab^ ± 28.46	90.81 ^abcde^ ± 7.53	12.10 ^b^ ± 0.18
OS2	1.34 ^a^ ± 0.05	198.92 ^abcde^ ± 20.87	97.62 ^bcde^ ± 1.51	14.31 ^f^ ± 0.26
OS3	1.27 ^ab^ ± 0.02	206.38 ^cdef^ ± 33.90	75.90 ^a^ ± 5.71	13.43 ^d^ ± 0.22

Data are presented as mean ± SD of three package-level experimental replicates (n = 3). Different superscript letters within the same column indicate statistically significant differences among treatment combinations at *p* < 0.05, as determined by multifactorial ANOVA followed by Tukey’s post hoc test. C—control sample, day 0; Cp—standard macro-perforated plastic packaging; OS0—chitosan:alginate, 1:0.25 (*w*/*w*) film; OS2—chitosan:alginate:turmeric, 1:0.25:2 (*w*/*w*) film, OS3—chitosan:alginate:oregano, 1:0.25:1 (*w*/*w*) film.

**Table 3:** Interactions of packaging type, storage temperature, and time on the total viable count (ANOVA).

Factor	Significance
Packaging type (PT)	n.s.
Temperature (T)	**
Period (P)	**
PT × T	n.s.
PT × P	**
T × P	**
PT × T × P	**

Significant interactions at *p* < 0.01 are indicated by **, “n.s.” denotes non-significant effects (*p* > 0.05).

## Data Availability

All data are contained within this article and are available upon request.
